# Surface Modification of Spruce and Fir Sawn-Timber by Charring in the Traditional Japanese Method—Yakisugi

**DOI:** 10.3390/polym13101662

**Published:** 2021-05-20

**Authors:** David Hans Ebner, Marius-Catalin Barbu, Josef Klaushofer, Petr Čermák

**Affiliations:** 1 Department of Wood Science and Technology, Faculty of Forestry and Wood Technology, Mendel University in Brno, Zemědělská 3, 613 00 Brno, Czech Republic; xebner@mendelu.cz; 2 Forest Products Technology & Timber Construction, Salzburg University of Applied Sciences, Markt 136a, 5431 Kuchl, Austria; marius.barbu@fh-salzburg.ac.at (M.-C.B.); pivopro@gmail.com (J.K.); 3 Faculty for Design of Furniture and Wood Engineering, Transilvania University of Brasov, B-dul. Eroilor nr. 29, 500036 Brasov, Romania

**Keywords:** surface charring, surface modification, thermal treatment, wood char, Yakisugi method, cupping effect

## Abstract

The traditional Japanese method of wood surface charring was studied. To perform the surface charring, three sawn Norway spruce and Silver fir wood boards of dimension 190 × 24 × 4000 mm^3^ were tied together to act as a chimney and charred in a short time (3–4 min) with open flame at a temperature above 500 °C. Temperature inside the chimney was recorded on the three different positions during the charring process. Surface temperature of spruce increased from 0 °C to 500 °C in approx. 120–300 s while fir increased in approx. 100–250 s. The thickness of the charred layer and the resulting cupping effect were investigated at the different heights of the chimney to evaluate its variability. Temperature achieved during the charring process was sufficient to get a significant charred layer of 2.5 and 4.5 mm on average for spruce and fir samples, respectively. The analyzed samples showed a significant cupping effect to the charred side with no difference between the annual ring orientation of sawn boards. Spruce exhibit a more significant cupping effect when compared to fir, i.e., 3.2–6 mm and 2.2–4.5 mm, respectively. Furthermore, the pH values of charred samples increased significantly, which could be an indication of improved resistance against wood-decay fungi. For better insight into the traditional charring method, further studies should be carried out to execute the charring process in a consistent quality and therefore fully exploit its potential.

## 1. Introduction

Nowadays, the forest and wood-working products industry has to meet new challenges in terms of sustainable wood protection, especially when it comes to house facades made of sawn-timber claddings. There are currently many scientific studies on the subject of preserving timber against various environmental impacts [1,2]. Wood modifications, such as thermal, chemical, or mechanical treatments, to improve the physico-mechanical properties are available [2,3]. In the area of surface modification by wood charring, applied temperature and time are key parameters to enhance surface performance [4,5]. Various scientific studies are currently dealing with the subject of one-sided surface charring to increase the natural durability of wooden facades with different positive results to the shelf-life duration or service time [6,7,8]. There is certainly no novelty about the idea of charring sawn wood for claddings. The traditional Japanese charring method, which is still used nowadays, could also meet the requirements of wood preservative for timber in the 21st century [2] and be used for facades as well as other architectural, handcrafted, or design elements. However, the knowledge about the traditional charring process and its resulting material properties have not been scientifically assessed yet and available information is rather limited. While some modern methods use a gas flame, an electric muffle furnace or a hot metal plate to achieve wood surface charring [5,6], the traditional Japanese manufacturing process, named “Yakisugi” or “Shou Sugi Ban”, differs through the spontaneous combustion of sawn wood boards, which are used to produce the charred layer. Nowadays, a contact heating system by hot plate used for one-sided surface charring of wood allows greater control of the process conditions and provide uniform charred surface at temperature range of 200–400 °C [4,5,6,7]. Recent studies proved that the protective properties of charring were influenced by the wood species (spruce and pine), the temperature and time of charring, and the evenness of treatment. The contact angle increased due to charring, but the water uptake decreased only in spruce sapwood; pine did not experience the same effect [4]. The study was followed up by work on the sorption characteristics of the charred surface [7], and research proved that charring notably reduced liquid water and water vapor sorption, and reduced hygroscopicity, while chemical and dimensional stability depended on heating time and temperatures. The charred surface modified using hot plate method is uniform, less brittle, and non-coloring when touched, when compared to the traditional Japanese process.

In order to achieve proper charring of wood, four temperature phases should be fulfilled. The first phase of charring is drying the wood at 100 °C to a 0% moisture content (MC). During the first phase, wood dries, beginning with the loss of free water and finishing with bound water. During this phase, wood cell walls shrink which may cause microcracks and compressions in the weakest regions between the S_1_ and S_2_ cell wall layers [9]. During the second phase, when the wood is dry and heated up to around 280 °C, it begins to spontaneously break down to produce charcoal plus mixed gases and vapors together with some tar. The hemicelluloses and lignin components are pyrolyzed in the ranges 150–300 °C and 225–450 °C, respectively [7,8]. In the third phase, the intensive generation of flammable volatiles extracts from wood occurs from 300 to 450 °C. In this range, the depolymerization of the cellulose starts in the range of 300–350 °C. Finally, the carbon-carbon linkage between lignin structural units is cleaved from 370 to 400 °C [10]. The degeneration of lignin is an exothermic reaction, which peaks between 225 °C and 450 °C. All wood components end their volatile emissions at a temperature around 450 °C. In phase four at >450 °C, the remaining wood residue is char [10,11,12].

The aim of this work was to understand the Yakisugi traditional Japanese charring method performed using the domestic softwood species, i.e., Spruce (*Picea abies* (L.) Karst.) and Silver fir (*Abies alba* L.). The present study should address missing knowledge about how this traditional method can char domestic softwood species in a short time of temperature exposure and how these process parameters relate to the quality of charred surface. More specifically, the following questions should be addressed: (1) Will the thickness of the charred layer differ over the wood boards width and the chimney height? (2) Is there any significant difference between studied wood species, i.e., different thickness of charred layer or cupping effect?

## 2. Materials and Methods

Norway spruce (*Picea Abies* (L.) Karst.) and Silver fir (*Abies Alba* L.) wood boars from an Upper Austrian lumber mill (Sägewerk Dax KG, Salzburg, Austria) were studied. The wood boards were in grading classes III to IV according to the Austrian standard, pre-dried to an average MC of 12%. The spruce and fir sawn wood boards of dimension 190 × 24 × 4000 mm^3^ were used for charring process. All tests were carried out in winter conditions (−1 to +4 °C). In total, twelve test runs were completed with spruce (6 tests × 3 boards) and fir (6 tests × 3 boards) wood.

### 2.1. The Process of Timber Charring in the Traditional Japanese Method—Yakisugi

**Figure 1:**
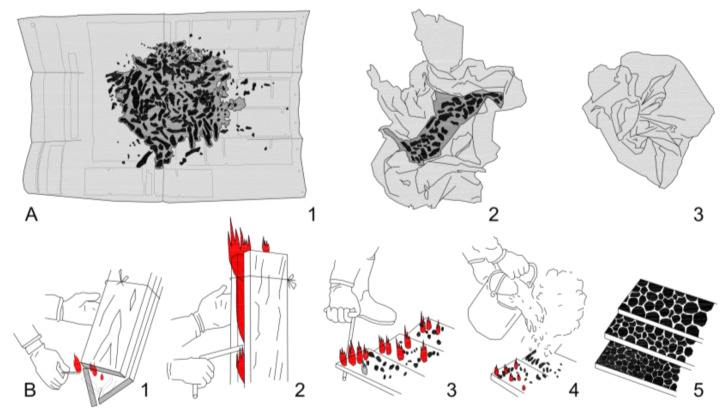
Guideline to handcraft an ignitor ball (**A**) and methodology of surface charring in the traditional Japanese method (**B**).

Sawn timber boards were one-sided surface charred by open fire using the traditional method. Three wood boards were tightened together using wet ropes, to create a triangle, to perform as a chimney. The orientation of the boards in a chimney, i.e., the bark or pith side, towards the flame was random. The starting energy was supported by a handcrafted ignitor ball (Figure 1(A3)). The ignitor ball consisted of a handful, roughly 50 g, of oakwood shavings, lightly compressed, wrapped in a newspaper size of 390 × 560 mm^2^ (Figure 1(A1–A3)). After inflaming the ignitor ball, the chimney provided enough energy to raise the internal temperature to inflame the wood. After lighting the ignitor ball, the ascending smoke flows through this chimney (Figure 1(B1)). In the beginning, the chimney was arranged inclined at approx. 70° to achieve a lower height where the ascending smoke runs easier from the bottom to the top. In this position, the chimney draft becomes stronger and reaches an internal temperature which allows the surface of the wood to burn. When the airflow sound is audible, the chimney is based in a vertical position. The ignitor ball falls down to the stone base of the chimney and continues burning. This helps for burning the lower edges of sawn wood. Without dropping down, the first 500 mm of the wood boards would not be charred in a proper way. When the right internal temperature is reached, the wood starts to burn on the inner side. The fire rises from the bottom to the top. During the first period of burning, the edges of the wood boards need to be switched. For this step, the rim-iron is used to give the wood a chance to get charred to the edges (Figure 1(B2)). When the fire hits the top of the boards and fire tongues reach out the gaps, where the wood boards touch each other, the charring process is completed.

After approx. 4 min, the burning process is stopped by flipping the chimney to the horizontal position. By releasing the ropes, the fire in the chimney stops immediately (Figure 1(B3)). The last glowing places get stopped with a soft water spray (Figure 1(B4)). As a result of this process, the wooden boards are fully covered by a 2–4 mm thick charred layer on the inner side of chimney (Figure 1(B5)). A great result should provide a uniform charred layer from one edge to the other, from the bottom to the top.

### 2.2. Surface Charring—Temperature and Time Measurements

**Figure 2:**
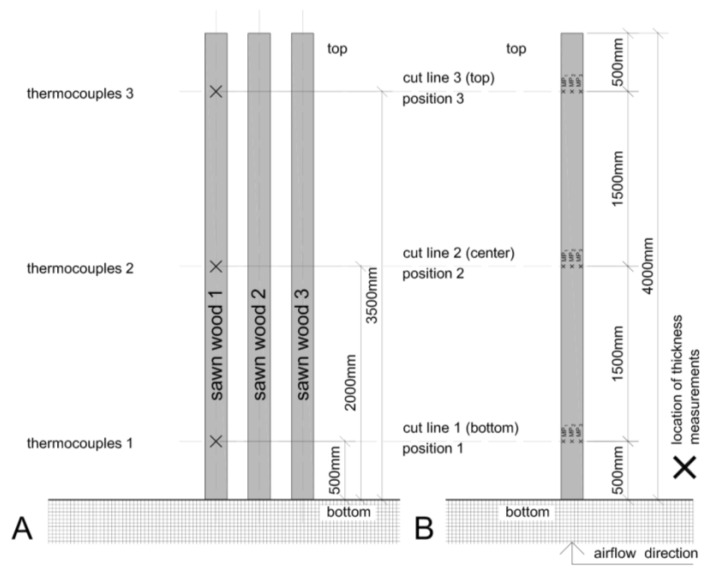
Position of thermocouples at different heights (**A**), cut lines for charred layer thickness (measurement points MP_1_, MP_2_, and MP_3_), and cupping effect measurements (**B**).

Temperature and time of the one-sided surface charring were measured on 12 repetitions, 6 times for spruce and fir wood boards. During the charring process, the temperature, which prevails inside the chimney, was monitored by using three NiCr-Ni temperature thermocouples (Type K). The thermocouples were placed into one of the three boards by drilling 8 mm holes perpendicular to the surface. The thermocouples were located in the middle of the samples at heights of 500, 2000, and 3500 mm, measured from the bottom of chimney (Figure 2A). Each of the thermocouples continuously displayed the temperature during the charring process on a digital display of the EMPlus 600 acquisition system (Eliwell, Italy).

Each charring process was continuously recorded. Simultaneously, the camera recorded the charring process, the increase of temperature, and time. The information about time is essential to determine the temperature increase inside the chimney in each section as shown in Figure 2A. The duration of each charring test required that in every section, i.e., positions 1–3, of the wood chimney the temperature needs to be higher than 400 °C for at least 60 s.

### 2.3. Charred Layer Thickness Measurement

The surface charred layer thickness was determined to evaluate the intensity of charring process and its variability at different positions within the boards. To measure the thickness of the charred layer the wood boards were cut into four pieces (Figure 2B). The cut lines were set according to the location of the temperature thermocouples (Figure 2A). Thus, the temperature values can be compared with the thickness of the charred layer. For the cutting procedure, the charred boards were turned around to avoid any charred layer damage. The thickness of the charred layer was measured with a digital caliper (PRECISE PS 7215, accuracy 0.01 mm). At each section line the thickness was measured three times at the points MP_1_, MP_2_, and MP_3_, as shown in Figure 2B.

### 2.4. Measurement of Cupping Due to the Charring

**Figure 3:**
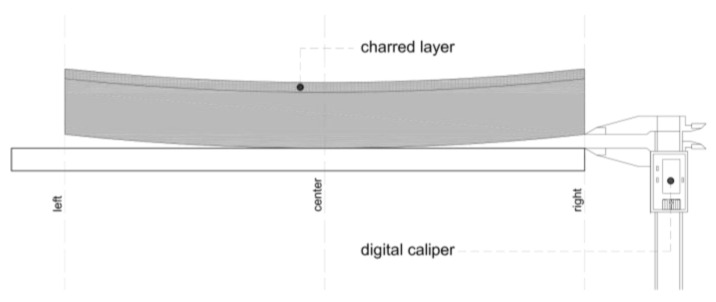
Cupping effect measurements of one-sided surface charred wood sample.

After the charring process, the cupping effect of the samples was determined with a digital caliper as shown in Figure 3. The measurements were carried out at the same samples which were used to measure the thickness of the charred layer.

The deformation was measured on the left and the right side of the sample, while placed in a restful way on a plane surface. The charred layer was on the upper side of the boards during these measurements.

### 2.5. pH Value Measurement

Four specimens were prepared to analyze the pH values of the charred layer. Two spruce and fir samples were analyzed according to the following procedure. The charred layer was removed with a laboratory spoon from the charred spruce and fir samples. Wood charcoal (approx. 1 g) from each sample was mixed together with 0.01 molar calcium chloride solution in a magnetic stirrer for about 60 min. After a rest period of 60 min, the suspension was tested with a laboratory pH-meter (HANNA Basic).

## 3. Results and Discussion

### 3.1. Charring—Temperature and Time

**Figure 4:**
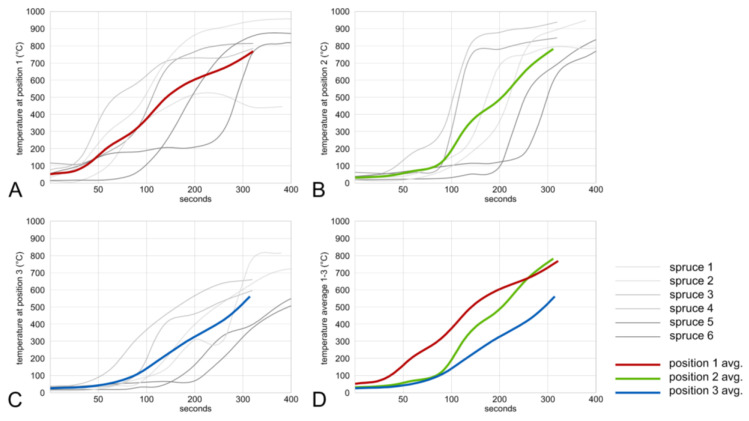
Temperature–time diagram for spruce samples at positions 1, 2, and 3 (**A**–**C**) and average temperatures (**D**).

**Figure 5:**
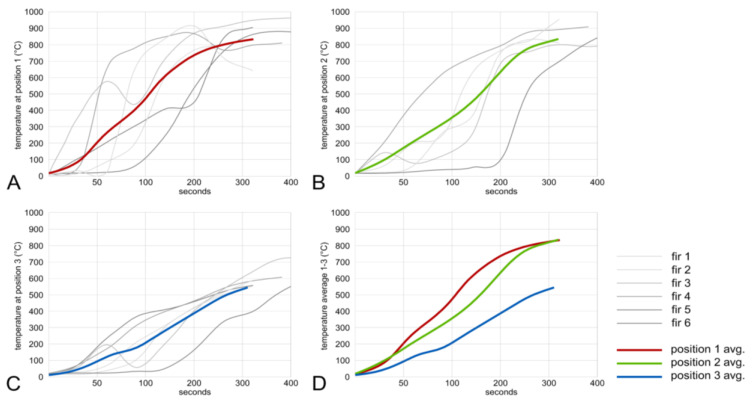
Temperature–time diagram for fir samples at positions 1, 2, and 3 (**A**–**C**) and average temperatures (**D**).

The charring test procedures were started by lightning up the ignitor ball. Time recording was started when smoke came out of the chimney. The time varied between 45 and 150 s and it is related to the initial MC of the boards [10], the quality of the ignitor ball, and some side effects, like wind and other factors. After the ignition ball has removed the residual moisture from the wood surface and raised the temperature to >280 °C, the flame starts to spread. The chimney effect provides sufficient oxygen to support the flame spread to increase the temperatures to perform all stages of the charcoal formation on the wood surface [11]. The temperature–time diagrams (Figure 4 and Figure 5) display the temperature increase at the three different locations, as shown in Figure 2A. All repetitions had an average charring time of 300 s. Temperature at position 1 increased first, while the lowest average temperature was measured at position 3. This behavior could be explained by the time required to dry the surface to 0% in the different positions of the chimney before the temperature reaches the point of ignition at around 300 °C. This temperature is reached, averaging all repetitions, with spruce in 84, 123, and 175 s for positions 1 to 3, respectively (Figure 4A–C).

Averaging all fir samples, repetitions reached the temperature of 300 °C in 67, 84, and 150 s for positions 1 to 3, respectively (Figure 5A–C). As can be seen in Figure 5, fir samples achieved the desired temperature (300 °C) much faster, when compared to spruce samples. When the flame spreads through the chimney, the charring process is easily audible. The temperature–time diagrams generally show the high variability in measured data. For instance, temperature can increase very fast, as shown by repetition no. 5, but also very slowly as shown by repletion no. 6 (Figure 5A). This can be influenced by many factors (initial MC, environment conditions), but also the nature of the burning process itself has a key impact on the consistent quality of charred wood. According to the literature, when the Yakisugi method is properly executed, temperature over 600 °C can be reached in about 30 s [13]. More repetitions of the charring process under steady conditions would be necessary to receive more substantial data and reveal the influence of these factors.

### 3.2. The Thickness of the Charred Layer

**Figure 6:**
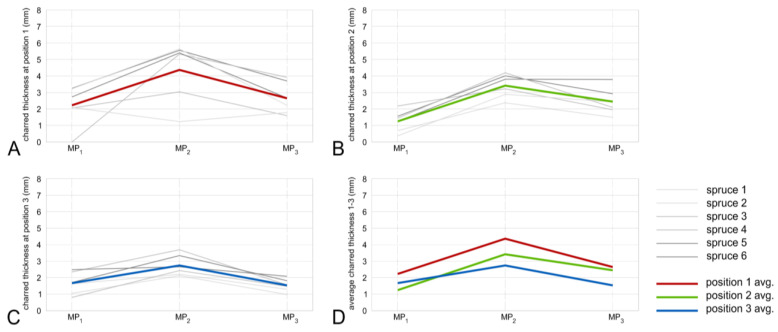
Spruce charred layer thickness in cutline 1—bottom (**A**), cut line 2—middle (**B**), cut line 3—top (**C**), and all average thicknesses (**D**).

**Figure 7:**
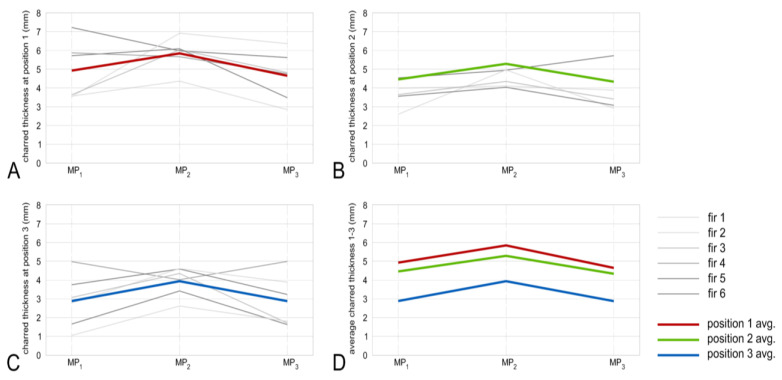
Fir charred layer thickness in cutline 1—bottom (**A**), cut line 2—middle (**B**), cut line 3—top (**C**), and all average thicknesses (**D**).

The results of thickness measurement show that the charred layer is thicker in the bottom part of charred boards with a significant decrease at the end (top of the chimney). This can be attributed to the higher temperature and longer exposure time of the bottom part of the measured samples. Stelzer (2017) described that the thickness of the charred layer applied on Japanese cedar (*Cryptomeria japonica*) produced with the traditional “Yakisugi” method, is about 3 to 5 mm [13], which is in agreement with the presented results. Related to the geometrical shape of the wood chimney, the charred layer is always thicker in the center position (MP_2_) when compared to positions MP_1_ and MP_3_, which are closer to the edges of the boards (Figure 6 and Figure 7).

According to the results, the charred layer thickness is closely related to the temperature reached during the charring process. The charred layer thickness of spruce boards at MP_2_ was on average 4.37, 3.42, and 2.74 mm at cutline positions 1 to 3, respectively (Figure 6D). Results of fir samples provide visually a more homogeneous charred layer when compared to spruce. The charred layer thickness of fir boards at MP_2_ was on average 5.84, 5.29, and 3.94 mm at cutline positions 1 to 3, respectively (Figure 7D). Basically, the higher temperature resulted in the thicker charred layer, however higher temperature does not always necessarily provide different thicknesses of the charred layer. This can be declared by the intensity of combustion, i.e., applied temperature. As mentioned in the literature, low temperature gains a higher yield of charcoal with low grade while higher temperatures generate charcoal of higher grade. For instance, at a carbonization temperature of 300 °C, 68% of the charcoal is fixed with 31% volatile material share. At a temperature of 700 °C, the yield is smaller but the percentage of fixed charcoal is 92% where the percentage of volatile materials is only 7% [12].

There is a significant difference in the observed results when spruce and fir are compared. Fir boards reached a higher temperature in a shorter time which resulted in a thicker charred layer when compared to spruce. Slightly different densities of studied wood species could contribute to the faster ignition and combustion of fir samples. The different density can also influence the thermal conductivity and the overall reaction to fire [14]. One-sided surface charring process of fir was notably faster and the charred surface was more homogenous when compared to spruce surface. These surface quality differences can be explained by the presence of the resin canals in spruce wood [15]. During the charring process, small explosions of resin canals are hearable which causes cracks in the charred layer on the surface of modified boards.

### 3.3. Cupping Caused by Surface Charring

**Figure 8:**
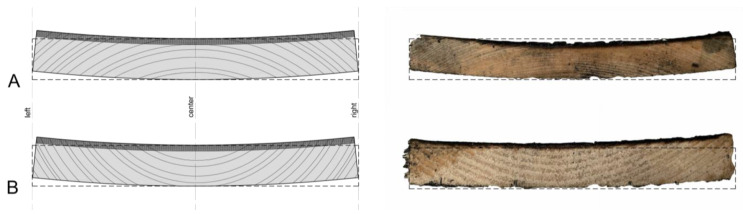
Illustration of the cross section (24 × 190 mm^2^) of spruce (**A**) and fir samples (**B**).

**Figure 9:**
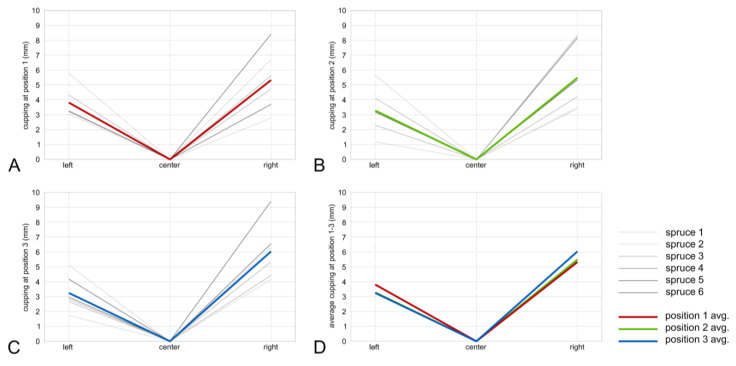
Spruce cupping in cutline 1—bottom (**A**), cut line 2—middle (**B**), cut line 3—top (**C**), and all averaged thicknesses (D).

**Figure 10:**
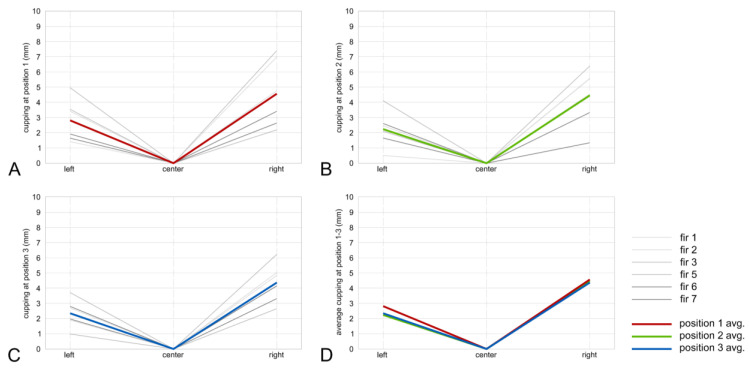
Fir cupping in cutline 1—bottom (**A**), cut line 2—middle (**B**), cut line 3—top (**C**), and all averaged thicknesses (**D**).

Photographs of charred samples were made from each face for the 36 section lines (Figure 2B). According to the results, all analyzed samples showed a cupping effect after the charring process. All charred samples from studied wood species had a cupping effect towards the charred layer side (Figure 8A,B). Spruce samples exhibit a more significant cupping effect when compared to fir samples, i.e., on average 3.2–6 mm and 2.2–4.5 mm, respectively (Figure 9 and Figure 10). Even though the higher temperature and therefore thicker charred layer was achieved for fir boards, the cupping effect was less significant. The explanation can be found in the minor different structural variation between spruce and fir boards as well as within a species itself [9]. Different density over the board’s thickness, orientation, width, and number of annual rings may play an important role in the cupping effect [9].

Naturally, the cupping effect is a result of different radial and tangential shrinkage of flatsawn timber. As a result of bound water removal in the range of 30–0%, the bark side of the board shrinks more significantly when compared to the pith side (closest to the center of the tree) as described by Virta (2005) [16].

However, according to Figure 8B, it is evident that the impact of open fire on the board’s surface has a stronger influence then the usual effect of wood cupping. Therefore, the position of the tree rings (pit/bark side) during the charring process has shown no influence on the cupping effect. It is hypothesized that this is caused by rapid temperature increase in the wood chimney and therefore moisture variation on both sides of charred board. The charred surface is much drier (0%) than the rest of the board (±12%) and therefore the cupping toward the pith side occurred. It is believed that charred layer has increased porosity, decreased density and volume, and does not exhibit swelling. Therefore, the board cupping remains after the conditioning to ambient relative humidity.

The charred layer on the boards surface works as an asymmetrical coating. As an example, wood materials should be coated on both sides to prevent eventual deformations. According to the Niemz and Sonderegger (2017) and Paulitsch and Barbu (2015), one-side coatings trigger deformations on the wooden composite related to the board, coating material thickness, moisture content, relative humidity, and the degree of asymmetrical coatings [17,18]. This can also be an argument for charred wood because solid wood is more inhomogeneous when compared to other wood-based materials. This may be a serious issue when the one-sided charred wood will be fixed as cladding for exterior use.

### 3.4. pH Value

Results show average pH values of 7.8 and 7.3 for spruce and fir, respectively. When comparing with the pH value of native wood, the pH value increased. According to Wagenführ (2007), spruce has a pH value of 4.0–5.3 and fir has a pH value of 5.5–6.1 [19]. The higher pH value of charred wood correlates with results published by Weber and Quicker (2018) who stated that pH value of carbonized wood varies between 4 and 12 [20]. The pH value matters when it comes to long lasting durability for outside application. The growing environment for the white rot fungi (*Stercum sanguinolentum*) and the brown rot fungi (*Phacolus schweinitzii*) is determined to be at the pH level between 4.5 and 5.5. Fengel and Wegener (1989) state that the optimum pH value for the brown rot fungi is 4.0 and for the white rot fungi it is between 3.7 and 4.2 [9]. These results could be an indication of improved resistance of one-sided charred wood against wood-decay fungi.

## 4. Conclusions

The traditional Japanese method of surface charring is an efficient way to modify the surface of selected domestic wood species. The chimney effect supplies enough oxygen which is necessary for wood surface charring and desired temperatures can be achieved in a relatively short time. The results showed that fir wood has some minor advantages over spruce wood. According to the results, fir boards reached high temperature in a shorter time as compared to spruce boards. The results also revealed that the charred layer is thicker in the bottom part of charred boards with a significant decrease at the end as a result of the higher temperature and longer exposure time of bottom part of the chimney. Furthermore, based on the geometrical shape of the wood chimney, the charred layer is always thicker in the center position when compared to the positions which are closer to the edges of the charred boards. Higher temperature reached during the charring process of fir boards resulted in the thicker charred layer and therefore a more significant cupping effect. Additionally, the absence of resin canals has a visual effect resulting in a more homogeneous charred surface of fir samples. The pH value of the studied wood samples was increased by charring process, which potentially indicates improvement in the natural durability against wood-decay fungi. The disadvantage of this traditional method is lack of any standard conditions and procedure. The nature of the traditional charring process can be affected by many parameters related to the weather conditions (wind, temperature), sawn wood (species, density, initial moisture content) as well as technical skills. Further research on traditional wood charring should be carried out in the future, in order to fully understand its potential for domestic wood species and reveal the missing knowledge about the process conditions and resulting material properties.
